# 

**Published:** 1903-09-15

**Authors:** 


					﻿THE
DENTAL REGISTER
EDITED BY
NELVILLE S. HOFF, D. D. S„
ANN ARBOR, MICH.
15, 1903.	No. 9.
PRICE, ONE DOLLAR A YEAR IN ADVANCE.
PUBLISHED MONTHLY BY
SAMIJEL· A. CROCKER & CO.,
THE OHIO DENTAL AND SURGICAL DEPOT.
3S to 30 W. “tli iSt., Ciiieiiiiiiiti, O.
Entered at the Postoffice at Cincinnati, 0., as second-class mail matter.
				

